# Fathers’ attachment representations and infant feeding practices

**DOI:** 10.1016/j.appet.2019.104374

**Published:** 2019-11-01

**Authors:** Samantha Reisz, Ashleigh I. Aviles, Serena Messina, Robbie Duschinsky, Deborah Jacobvitz, Nancy Hazen

**Affiliations:** a University of Cambridge, Public Health & Primary Care, UK; b University of Texas at Austin, Human Development & Family Sciences, USA

**Keywords:** Parent-infant feeding, Parental feeding behavior, Fathers, Attachment

## Abstract

This study examined how fathers' adult attachment representations, assessed before the birth of their first child, predict feeding practices with their 8-month-old infants. Fathers have been underrepresented in child feeding research, particularly in longitudinal and observational studies. Feeding is a key parenting task of infancy and a growing number of studies have begun to explore the connection between attachment and parental feeding practices and behavior, revealing a clear link between mothers' adult attachment and how they feed their children. This is the first longitudinal examination of attachment as a prenatal predictor of fathers' infant feeding behavior. Participants were 118 first-time fathers and their infants. Adult Attachment Interviews were conducted in the third trimester of pregnancy, and father-infant feeding interactions were observed at home when the infant was 8-months-old. Videotaped feedings were coded using Chatoor's Feeding Scale (1997). Compared to other fathers, (1) those with secure attachment representations were more attuned to their infants during feeding, (2) those with dismissing representations were less attuned, and (3) those with unresolved trauma displayed more controlling behaviors. Fathers were more controlling with their sons than their daughters across all attachment representations. Study results suggest that father's infant feeding behaviors may influence by their own attachment representations. The links to fathers' controlling feeding practices are noteworthy because of the negative implications controlling parental feeding practices can have on child outcomes. The prediction of paternal feeding behaviors from assessments conducted prenatally has important intervention implications.

## 1 Introduction

Fathers have become increasingly involved in the care of infants in western societies over the past several decades (Bianchi, 2000; Fraser et al., 2011), including feeding activities (Jones, 2013, p. 71; Mallan et al., 2014). Yet most studies of parental feeding practices with infants have been focused on mothers. Only a handful have addressed fathers' feeding practices and the influence of fathers' characteristics on their feeding behaviors (e.g., Blissett, Meyer, & Haycraft, 2006; Haycraft & Blissett, 2008; Khandpur, Blain, Fisher, & Davison, 2014). A review of 667 observational studies of parental feeding and child obesity reported that fathers represented 17% of participants across the studies included, and only 10% reported results of fathers specifically (Davison et al., 2016). Sample sizes of fathers were small in comparison with mothers, with 59% including fewer than 50 fathers. Moreover, only 1% of the 667 observational studies reviewed on parenting and childhood obesity included only fathers (Davidson et al, 2016). A review of paternal feeding practices by Khandpur, Blaine, Fisher, and Davison (2014) included 20 studies and noted that all but one study used a cross-sectional design, meaning that only correlates of feeding practices could be examined, rather than predictors. The studies that have examined effects of fathers' parenting, feeding practices, weight, and food behaviors on children's eating behaviors suggest that fathers may impact children independently of mothers (see Fraser et al., 2011 for a review of 10 studies). Thus, it is important to understand influences of fathers' feeding behaviors on their children. The current study employs a longitudinal design to address predictors of observed paternal feeding practices in infancy.

Application of theoretical models to understanding father feeding behavior have been limited to date. Only four of the 20 studies examined by Khandpur and colleagues' (2014) review included a theoretical model or framework to address their research questions. Calls have been made to utilize theoretical models to better situate paternal feeding practices in the larger literature on fatherhood and feeding generally (Khandpur et al., 2014). This study draws on attachment theory and fits into Cabrera, Fitzgerald, Bradley, & Roggman's (2014) ecological model of fatherhood. According to this model, fathers' personality and personal characteristics (including father's attachment representations) have bidirectional effects on fathers' parenting behaviors (including feeding), which, in turn, affect their children's development. An attachment perspective may be particularly useful in understanding the roots of fathers' feeding practices during infancy. Parents' attachment representations are a powerful predictor of their caregiving behaviors (George & Solomon, 2008), and a small but growing number of studies have begun to explore attachment as a lens for understanding parental feeding practices and behavior (e.g., Bost et al., 2013; Messina, Reisz, Hazen, & Jacobvitz, in press; Pickard, Townsend, Caputi, & Grenyer, 2017; Powell, Frankel, Umemura, & Hazen, 2017). Feeding is a primary parenting task during the first year of life. As such, it provides a fundamental context in which to observe the caregiver-infant relationship (e.g., Ainsworth, 1984; Egeland & Farber, 1984). According to attachment theory, it is during such repeated interactions that caregivers learn to interpret infant communications about their needs and emotions (Ammaniti, Lucarelli, Cimino, D’Olimpio, & Chatoor, 2011; Egeland & Brunnquell, 1979), and infants come to trust the degree to which their caregiver will be available and responsive (Ainsworth, 1979; 1984). Although fathers' parenting styles have been associated with their feeding practices (Collins, Duncanson, & Burrows, 2014; Pratt, Hoffman, Taylor, & Musher-Eizenman, 2017), to the authors' knowledge, this is the first study to examine fathers' attachment security as a longitudinal predictor of observed father-infant feeding behaviors.

The ability of caregivers to attune to infants' emotional states during feeding is particularly important because infants can begin meals in a state of distress due to hunger. The caregiver's task is simultaneously to modulate infant arousal and effectively feed them. This asks that the caregiver to be attuned to the child's emotional cues. Attunement between infant and caregiver during feeding is an infant-centered strategy that supports infants' developing capacity to identify their own hunger and satiety cues, helping them to develop feeding autonomy (Black & Aboud, 2011) and to learn healthy, self-regulated eating habits that protect against overweight and obesity (Birch & Ventura, 2009; Frankel et al., 2012).

In contrast to attunement, controlling parental feeding behaviors are parent-centered strategies, which serve the parent's goals and may not take into account the child's emotional or psychological needs (Vaughn et al., 2016). Fathers may engage in controlling behaviors when feeding infants, particularly in response to slow infant eating (Haycraft & Blissett, 2012; Mallan et al., 2014). Multiple studies provide evidence that fathers may be more controlling during feeding than mothers, often using coercive feeding behaviors and pressuring children to eat (Brann & Skinner, 2005; Powell, Frankel, Umemura, & Hazen, 2017; Pratt et al., 2017; Pulley, Galloway, Webb, & Payne, 2014), although other studies have not found such differences (Blissett et al., 2006; Vollmer, Adamsons, Foster, & Mobley, 2015). Controlling feeding behaviors can interfere with the development of a child's understanding of their own internal cues for hunger and satiety (Golan & Bachner-Melman, 2011; Patrick, Hennessy, McSpadden, & Oh, 2013) and have been linked to poor health outcomes in children (Birch, Fisher, & Davison, 2003; Blissett, Haycraft, & Farrow, 2010). Paternal controlling feeding behaviors have also been linked to higher food avoidance in their children (Vollmer et al., 2015). It is important to understand predictors of parents' controlling feeding behavior because of their connection to poor child outcomes, such as increased risk of overweight and obesity (Frankel et al., 2012) and behavior problems (Hemmi, Wolke, & Schneider, 2011), including connections that may be specific to fathers.

Attachment theory asserts that caregivers' availability and responsiveness to their children is influenced by mental representations of their relationships with their own caregivers during childhood (Bowlby, 1980; George & Solomon, 2008; Main, Kaplan, & Cassidy, 1985). The gold-standard method of assessing representations of attachment in adulthood is the Adult Attachment Interview (AAI; George, Kaplan & Main, 1985). Caregivers who can discuss childhood relationships with their parents in an open, objective, and believable way are classified as secure. These caregivers have been found to be able to accurately perceive their children's distress cues and respond effectively (Van IJzendoorn, 1995; George & Solomon, 2008). In the context of feeding, this should result in parents showing greater sensitivity to their infants' feeding, hunger, and satiety cues, such as an infant turning away from food that is being offered. It should also relate to greater overall emotional attunement, leading to a more positive and enjoyable feeding session.

Caregivers who cannot discuss their childhood parental relationships coherently are classified as insecure-dismissing or insecure-preoccupied. These caregivers often struggle to respond consistently and sensitively to their children's distress (Adam, Gunnar, & Tanaka, 2004; Pederson, Gleason, Moran, & Bento, 1998; Van IJzendoorn, 1995). Dismissing attachment representations involve minimizing the importance of early negative experiences and attachment relationships (Main, Goldwyn, & Hesse, 2002). In feeding situations, this may lead to emotional disengagement and a “hands-off” attitude. In contrast, preoccupied representations are characterized by an enmeshment in past relationships with parents, which can take the form of angrily blaming their parents for wrongdoings, in the context of the AAI (Main et al., 2002). Infant negative affect, including distress or irritation stemming from hunger, has the potential to activate these fathers' own attachment representations. This may affect both fathers' accuracy in perceiving their infants' cues, as well as their capacity to respond sensitively. During feeding, this could result in conflict throughout the interaction and attempts to control infant behavior. Indeed, Messina et al. (2019) found that preoccupied maternal attachment representations, assessed prenatally, predicted higher levels of conflict and control, and lower levels of attunement, during feeding. Fathers who are dismissing may similarly struggle to accurately perceive and respond to infant feeding cues, displaying less attunement and more conflictual and controlling feeding practices with their infants than fathers who are secure.

In addition to organized attachment representations (i.e., secure, dismissing, and preoccupied), adults can also be classified as *unresolved for trauma stemming from experiences of loss or abuse* on the Adult Attachment Interview if they display signs of mental disorganization when discussing experiences of loss or abuse (Jacobvitz & Reisz, 2018; Main et al., 2002). Unresolved trauma is a particularly strong risk factor that can affect caregivers' capacity to attune to their infants and effectively respond to their needs (Solomon & George, 2011; Jacobvitz, Leon, & Hazen, 2006). Unresolved trauma in fathers specifically has been linked to hostile and role-reversed caregiving during home observations of father-infant interactions (McFarland-Piazza, Hazen, Jacobvitz, & Boyd-Soisson, 2012). Mothers classified as unresolved are more likely to have an infant classified as failure-to-thrive than mothers who are not unresolved (Ward, Lee, & Lipper, 2000), suggesting that unresolved trauma in the parent can result in problematic infant feeding patterns. Furthermore, Messina et al. (2019) found that unresolved attachment in mothers predicted their use of controlling behavior during feeding. It follows that fathers' unresolved trauma may also influence controlling behavior during feeding interactions. If fathers’ memories of unresolved trauma become activated within the context of feeding, such as by signs of infant distress, fathers may strive to regain a sense of control through the use of controlling feeding practices.

Most studies that have examined links between adult attachment and parents' feeding practices have used self-report measures of parents' attachment style (e.g., Bost et al., 2013; Pickard et al., 2017; Powell et al., 2017), which is conceptually similar though not identical to attachment representations assessed using the AAI (Crowell, Fraley, & Roisman, 2016; Ravitz, Maunder, Hunter, Sthankiya, & Lancee, 2010). Pickard et al. (2017) found that mothers' self-reported secure attachment style related to greater observed maternal responsiveness during feeding at 7 and 10 weeks postpartum. Bost and colleagues (2013) found that caregivers with insecure attachment styles reported using emotional and pressuring feeding practices with their children. However, 90% of the sample was mothers, and no differences between mothers and fathers were reported. Furthermore, neither of these studies examined differences between types of attachment insecurity. Powell and colleagues (2017) found that mothers and fathers’ self-reports of higher levels of attachment anxiety, which is similar to preoccupied attachment strategies assessed by the AAI, were related to reports of higher levels of controlling feeding behavior with preschoolers. This relation was not seen in those with higher levels of attachment avoidance, though, which is similar to dismissing attachment. It is notable that 32% of the parents assessed were fathers and the relationship between attachment style and feeding was similar for mothers and fathers. The difference in findings between anxious and avoidant attachment styles found by Powell and colleagues (2017), and between preoccupied and dismissing attachment representations found by Messina et al. (2019), demonstrates the importance of examining insecure categories separately, given their associations with different feeding practices. This study examined all insecure categories separately, including unresolved because of its prior connections to controlling feeding practices.

Literature on parental feeding practices indicates that it may be important to take into account child-related factors, such as sex or gender, when exploring the antecedents of parents' feeding practices, as parents may behave in different way towards their daughters and sons. This differential treatment may be related to parents’ assumptions about gender based on child sex and stereotypes regarding optimal body sizes of boys versus girls. For example, one study found that both mothers and fathers of toddlers were more likely to view their sons as underweight, but not their daughters (Holm-Denoma et al., 2005). Another study found that fathers used pressuring tactics with their sons four times more frequently than with their daughters (Orrell-Valente et al., 2007). Thus, it is possible that fathers in this study may employ different feeding practices with sons and daughters.

### 1.1 Present study

The present study is the first to longitudinally examine connections between fathers' attachment representations, assessed prenatally using the Adult Attachment Interview (George, Kaplan, & Main, 1985; 1996), and their observed feeding practices with their 8-month-old infants. Eight months is an ideal time in a child's life to observe father-infant feeding interactions. In general, American fathers become increasingly involved in infant feeding after the transition to solid foods, especially if mothers have been breastfeeding (Thullen, Majee, & Davis, 2016). At this age, infants may also be beginning to experiment with self-feeding, allowing for the observation of a larger range of parental behaviors in terms of attunement, conflict, and control. Video-recorded interactions between fathers and children are rare in the literature, as the majority of father-child research to date has been dominated by survey and self-report methodology (Cabrera, Volling, & Barr, 2018). Observational research on parent-infant feeding is particularly important since parents may lack awareness of how they feed their infants, and self-reports may be further biased by social desirability. Four hypotheses were tested: (1) Fathers' secure attachment representations would predict higher attunement and less conflictual and controlling behaviors during feeding; (2) fathers' dismissing attachment representations would predict lower attunement during feeding; (3) fathers' preoccupied attachment representations would predict less attuned and more conflictual and controlling behaviors during feeding; and (4) fathers' unresolved trauma would predict more controlling behaviors during feeding.

## 2 Method

### 2.1 Participants

Participants were 118 fathers and their infants. One-hundred-twenty-five heterosexual couples expecting their first child were recruited during pregnancy through childbirth classes, public service radio announcements, and flyers distributed at local maternity stores and obstetricians’ offices in the larger geographical area surrounding Austin, Texas, USA. All parents were either married or living together at the start of the study. Median paternal age was 30 years, ranging from 19 to 51. The majority of families were middle class, though income ranged from the poverty line to upper-middle class. Median income for the sample was $30,000–$44,999. Of the 125 families in the sample, 17% earned less than $30,000, 24.3% earned $30,000–$45,000, 27.1% earned $45,000–$60,000, and 31.4% earned over $60,000. The participants were generally well educated: 8% of the fathers earned a high school degree, 34% had some college or trade school training beyond high school but did not graduate from college, 38% earned a bachelor degree, and 17% had a graduate or post-college degree. Eighty-two percent of participants were white, 9% were Hispanic, 3% were African American, and 6% were Native American, Middle Eastern, or Indian. All infants (41% female) were born full-term and none were admitted to the Neonatal Intensive Care Unit. When infants were 8 months old, 118 families were still participating in the study. There were no demographic differences between families that remained or left the study at that time.

### 2.2 Procedure

When their partner was in the third trimester of pregnancy, fathers completed the Adult Attachment Interview in a laboratory visit, and completed a series of questionnaires, including a demographic questionnaire during a home visit. When their child was 8-months-old, home visits were conducted in which each parent participated in multiple caregiving tasks with their infants, including a clothing and diaper change, free play, and feeding their infant. Feedings were counterbalanced, with one parent beginning the feeding and the other parent taking over halfway through. Feeding interactions were videotaped for later coding.

### 2.3 Measures

*Adult Attachment Interview* (AAI; George, Kaplan & Main, 1985; Main et al., 2002). The AAI is a semi-structured interview used to assess adult attachment representations. The interview lasts approximately one hour and is audio-recorded for later transcription and verbatim coding. Participants are asked to describe their relationship with their parents in childhood and evaluate the effects of their early experiences on their current personality and relationships. They are also asked to describe and evaluate several attachment-related experiences, including loss of attachment figures through death and threatening experiences like abuse.

Participants were first classified into one of three organized categories: autonomous, dismissing, or preoccupied. Autonomous states of mind are characterized by an ability to coherently describe childhood relationship experiences in a manner that is clear, believable, and truthful, independent of whether those experiences were positive or negative. Dismissing states of mind are characterized by an insistence upon an inability to remember childhood experiences, idealization of one or both parents, and derogation of their attachment figures and attachment generally. Preoccupied states of mind are characterized by involving anger towards one or both parents and/or passive discourse that is vague, difficult to keep track of, and can slip into childlike phrasing or linguistic confusions between themselves and their parent. Interviews are coded for unresolved states of mind with respect to loss or abuse in addition to the three primary organized classifications, and the unresolved classification can be analyzed as a separate category. Unresolved states of mind are characterized by brief lapses in the monitoring of discourse and/or monitoring of reasoning while discussing traumatic experiences stemming from abuse and/or loss. Indicators of unresolved loss include lapses in the monitoring of reasoning, such as using the present tense to discuss someone who is dead, and lapses in the monitoring of discourse, such as a marked change in coherence uncharacteristic of responses while discussing that loss. Indicators of unresolved trauma include lapses in the monitoring of reasoning, such as unsuccessful attempts to deny the abuse and/or fears of being mentally possessed by the abuser.

In this sample, 53 fathers were classified as secure, 35 were dismissing, 7 were preoccupied, and 23 were unresolved. All interviews were coded by two graduate students who had successfully completed training and were certified to code the AAI. One of the students coded all of the AAIs and the second coded 28% of the transcripts for reliability (N = 35). Exact agreement between the two coders on the four-way AAI classification – secure, dismissing, preoccupied, and unresolved – was 88% (k = 0.85).

The AAI is a reliable assessment tool that has demonstrated both discriminant validity (e.g., Bakermans-Kranenburg & Van IJzendoorn, 1993; 2009; Sagi et al., 1994) and predictive validity (e.g., Fonagy, Steele, & Steele, 1991; Van IJzendoorn, 1995). AAI classifications have been shown to be independent of verbal and performance IQ, social desirability, personality, autobiographical memory for other topics, and narrative style when discussing other topics (Bakermans-Kranenburg & Van IJzendoorn, 1993; Sagi et al., 1994; Van IJzendoorn, 1995).

*Feeding Scale* (Chatoor et al., 1997). The Feeding Scale is an observational assessment tool that assesses the quality of the caregiver-infant feeding interaction. The scale includes 46 items that assess caregiver and infant behaviors on a Likert-type scale ranging from “0 - behavior does not occur” to “4 - Behavior occurs extremely often”. Chatoor used the 46 items to create 5 subscales: *Dyadic Reciprocity, Dyadic Conflict, Talk and Distraction, Struggle for Control, and Maternal Non-Contingency.* The internal consistency of the original Chatoor scales showed alphas that were below acceptable levels for this sample, so items were rearranged conceptually to create three subscales that were used in this study: *Attunement* (e.g., “Waits for infant to initiate interactions”; α = 0.76), *Conflict* (e.g., “Makes negative statements about infant's food intake or preferences”; α = 0.77), and *Control* (e.g., “controls feeding by overriding infant's cues”; α = 0.67). Each subscale includes items for parent and child behavior. For the purposes of this study, only the parent items were used to specifically examine fathers' feeding practices.

The Feeding Scale has been established as a valid and reliable assessment tool (Chatoor et al., 1997; Lotzin et al., 2015). Its construct validity was first established in a study with 74 infants and toddlers with feeding disorders and 50 non-clinical comparisons, and discriminant validity was established between the clinical and non-clinical groups (Chatoor et al., 1997). For the present study, two trained coders blind to the other study variables coded all father-infant feeding interactions. Inter-rater reliability was k = 0.78 for Attunement, k = 0.78 for Conflict, and k = 0.68 for Control. While the subscales used in this study differ from Chatoor's original scales, the items are the same, and the internal consistency and interrater reliability of our conceptually derived scales suggest that they remain effective tools for assessing father feeding behavior.

## 3 Results

### 3.1 Preliminary analyses

Correlations between variables of interest and demographics can be found in Table 1. Father's age and education correlated with father feeding behaviors and thus were included as controls. Meaningful differences in fathers' feeding practices based on their education level have emerged in prior studies (e.g., Khandpur et al., 2014; Khandpur, Charles, Blaine, Blake & Davison, 2016; Zarnowiecki, Dollman, & Parletta, 2014), further supporting the inclusion of education as a covariate.

**Table 1:** Zero-order correlations of study variables.

Variable	1	2	3	4	5
1. Paternal age	–				
2. Paternal education	.30**	–			
3. Feeding attunement	-.26**	-.20*			
4. Feeding conflict	.03	-.02	.00	–	
5. Feeding control	-.14	-.18	.45***	.21*	–
*N*	123	126	113	113	111
*M*	31.6	4.47	.14	1.97	.41
*SD*	6.25	1.16	.10	.25	.28

Note: *p < .05; **p < .01; ***p < .001.

Difference tests were run to assess categorical differences across child sex and feeding order. There was a significant difference across all of fathers in the sample based on child sex, such that all fathers were more controlling with their sons than their daughters regardless of attachment, (*t*(108) = 2.34*, p = *.02). This difference was not present for feeding attunement, (*t*(110) = -0.23*, p = *.82), or feeding conflict, (*t*(110) = 0.54*, p = *.59). Feeding order was related to differences in father attunement during feeding, (*t*(109) = 2.61*, p = *.01), with fathers who fed the child first showing higher attunement than fathers who fed the child second. There were no feeding order differences for conflict, (*t*(109) = 1.23*, p = *.22), or control, (*t*(110) = 1.52*, p* = .13). Based on these findings, child sex and feeding order were included as control variables in all regression analyses.

### 3.2 Hypothesis testing of relations between father attachment and feeding behavior

Hypotheses were tested using a series of hierarchical regressions to assess whether fathers' Adult Attachment Interview classifications, measured prenatally, would predict their feeding behavior with their 8-month-old infants. The outcome variables for the sets of regressions were fathers' emotional attunement, conflict, and control, respectively. For each regression, the first step included the control variables: father age, education, feeding order, and child sex. The second step included dichotomous variables indicating the father's attachment classification, such that 1 indicated the classification being assessed and 0 indicated all other classifications.

*Secure representations.* As shown in Table 2, fathers’ secure (versus insecure) attachment classifications predicted higher attunement while feeding their infant at 8-months (β = 0.20, *p* < .05). Secure representations did not predict conflict or controlling feeding behavior.

**Table 2:** Hierarchical regression analyses for feeding behaviors in fathers who are secure.

Variable	Feeding Attunement		Feeding Conflict		Feeding Control	
	β	*p*	β	*p*	β	*p*
Feeding order	-.18	.06	-.13	.21	-.09	.38
Child sex (1 = female, 0 = male)	-.02	.82	-.03	.73	-.22*	**.02**
Paternal age	-.16	.11	.07	.50	-.06	.59
Paternal education	-.12	.23	-.05	.67	-.13	.21
Attachment (Secure)	-.20*	**.04**	.03	.81	-.14	.16
$R^{2}$	.17		.02		.12	
Δ*F* for $R^{2}$	4.22*		.06		2.05	

*Note. N* = 118. *p < .05; **p < .01; ***p < .001.

*Dismissing representations.* As shown in Table 3, fathers’ dismissing attachment classifications predicted lower attunement during feeding (β = −0.21, *p* < .05) compared to all other classifications (i.e., secure, preoccupied, and unresolved). Dismissing representations did not predict conflict or controlling feeding behavior.

**Table 3:** Hierarchical regression analyses for feeding behaviors in fathers who are dismissing.

Variable	Feeding Attunement		Feeding Conflict		Feeding Control	
	β	*p*	β	*p*	β	*p*
Feeding order	-.20*	**.04**	-.13	.19	-.12	.23
Child sex (1 = female, 0 = male)	.04	.63	-.03	.73	-.20*	.05*
Paternal age	-.16	.10	.07	.51	-.07	.49
Paternal education	-.13	.17	-.05	.64	-.16	.11
Attachment (Dismissing)	.21*	**.02**	-.09	.40	-.07	.50
$R^{2}$	.17		.03		.06	
Δ*F* for $R^{2}$	5.33*		.74		.46	

< 0.1; *p < .05; **p < .01; ***p < .001.

*Note*: N = 118 *p < .05; **p < .01; ***p < .001.

*Preoccupied representations*. As shown in Table 4, fathers’ preoccupied attachment was not significantly related to feeding interactions. Preoccupied representations did not predict attunement or controlling feeding behavior.

**Table 4:** Hierarchical regression analyses for feeding behaviors in fathers who are preoccupied.

Variable	Feeding Attunement		Feeding Conflict		Feeding Control	
	β	*p*	β	*p*	β	*p*
Feeding order	-.22*	**.02**	-.12	.25	-.11	.26
Child sex (1 = female, 0 = male)	.06	.55	-.04	.66	-.20*	**.04**
Paternal age	-.17	.08	.08	.45	-.07	.52
Paternal education	-.15	.12	-.05	.66	-.16	.13
Attachment (Preoccupied)	-.06	.53	.18	.07	.01	.91
$R^{2}$	.13		.05		.10	
Δ*F* for $R^{2}$	.40		3.41		.01	

*Note. N* = 118. *p < .05; **p < .01; ***p < .001.

*Unresolved representations of trauma due to loss or abuse.* As shown in Table 5, fathers’ unresolved attachment classifications predicted controlling behavior while feeding their 8-month-olds (β = 0.26, *p* < .05). Unresolved representations did not predict attunement or conflict during feeding.

**Table 5:** Hierarchical regression analyses for feeding behaviors in fathers who are unresolved.

Variable	Feeding Attunement		Feeding Conflict		Feeding Control	
	β	*P*	β		β	
Feeding order	-.22*	**.03**	-.13	.21	-.09	.33
Child sex (1 = female, 0 = male)	.05	.59	-.03	.70	-.24**	**.01**
Paternal age	-.17	.09	.07	.49	-.06	.54
Paternal education	-.15	.13	-.05	.65	-.12	.23
Attachment (Unresolved)	.02	.84	-.05	.63	.26**	**.01**
$R^{2}$	.13		.02		.16	
Δ*F* for $R^{2}$	.04		.23		7.50**	

*Note. N* = 118. *p < .05; **p < .01; ***p < .001.

## 4 Discussion

This study explored links between fathers' attachment representations, assessed prenatally using the Adult Attachment Interview (George, Kaplan, & Main, 1985/1996), and fathers' infant feeding practices. Calls have been made for more longitudinal and observational research on fathers' feeding practices (e.g., Khandpur et al., 2014; Penilla et al., 2017). The current longitudinal design begins to address this call, demonstrating that fathers' feeding practices can be predicted prenatally using their attachment representations. Father-infant interactions during feeding may hold implications for the developing father-infant relationship (Cabrera, Fitzgerald, Bradley, & Roggman, 2014), and have been linked to children's later health outcomes, including overweight and obesity (Frankel et al., 2012), general behavior problems in toddlerhood (Hemmi et al., 2011), and anxiety in middle childhood (Messina, 2016). To our knowledge, this is the first study that has specifically focused on fathers' attachment representations and their observed feeding behaviors. Results showed that fathers' attunement, conflict, and controlling behavior while feeding their 8-month-old infant could be predicted by their attachment representations that were assessed before the infant was born. This highlights the importance of considering attachment as a relevant antecedent of fathers' feeding practices.

Two strengths of this study are that it was grounded in an explicit theoretical context and employed a longitudinal design, which has been limited in the paternal feeding literature to date (Khandpur et al., 2014). Results demonstrated that different types of caregiver attachment representations predict different feeding patterns in theoretically predictable ways. This attachment framework complements Cabrera et al. (2014) expanded ecological model of fatherhood, which highlights how fathers' histories and personal characteristics influence their caregiving behavior, which subsequently predict child outcomes. The attachment perspective on feeding used in this study fits well into this model of fatherhood, highlighting the predictive role of fathers’ attachment representations on their feeding practices with their infants.

Fathers with secure attachment representations showed higher levels of attunement to their infants during feeding, as hypothesized. Parents classified as secure have consistently been documented as able to accurately perceive their children's cues and effectively respond to their children's distress (Van IJzendoorn, 1995; George & Solomon, 2008). The current findings are consistent with this view, showing that fathers who are secure can attune to their infants' needs during feeding. A substantial proportion of research on father-infant relationships has focused on play contexts, leaving a gap in our understanding of fathers in caregiving contexts such as feeding (Cabrera et al., 2018; Tamis-LeMonda, 2004). This is an important research gap to address because sensitive responsiveness to the infant's cues during feeding can help the child develop healthier eating habits based on their own satiety cues, which should help prevent overweight and obesity (Birch & Ventura, 2009; Black & Aboud, 2011). Further, parents who engage in responsive feeding practices support children's developing ability to self-feed, encourage them to experience new tastes and textures, and help children learn that eating and mealtimes are fun (Black & Aboud, 2011).

Dismissing attachment representations predicted lower attunement during infant feedings, consistent with the second hypothesis. Adults classified as dismissing tend to minimize the importance of attachment relationships and often rely on deactivating strategies in the face of stressful situations, including diverting attention away from the source of their distress and suppressing negative emotional responses (Hesse, 2016). For fathers with dismissing attachment representations, attunement to their infants' emotional states, which can include hunger and satiety cues, may represent a threat to the fathers' own emotional regulatory process of suppressing negative emotions if the infant is distressed. In the context of feeding, these behavioral tendencies may impair fathers' capacity to attune to their infants' hunger satiety cues. This could then lead them to ignore or override infants' cues, which can cause distress and interfere with the infants’ developing ability to understand their internal self-regulatory cues (Birch & Ventura, 2009; Black & Aboud, 2011). Interestingly, while fathers who were dismissing showed lower attunement than all other fathers, they did not display significantly more conflict or control. This distinction between a lack of attunement and genuinely conflictual behaviors is meaningful because of the possibility of different predictive pathways and resulting trajectories. Future research should continue to examine different categories of parental feeding behaviors to assess how meaningful such differences may be for child outcomes and intervention efforts.

Contrary to hypotheses, there was not sufficient evidence to support the hypothesized links between fathers’ preoccupied attachment representations and higher feeding conflict and lower feeding attunement. Past research has found that maternal preoccupied attachment representations significantly predicted conflict behavior during feeding (Messina et al., 2019). While this study did not find the same pattern for fathers, there was a marginal association linking father preoccupation with conflictual feeding behaviors. Only seven fathers in this sample were classified as preoccupied, though, limiting the power of the analysis. Thus, it is unclear whether the same pattern can be observed in fathers as in mothers. Further research with larger samples of preoccupied fathers is needed to assess whether there are meaningful similarities or differences between mothers and fathers in how preoccupied adult attachment representations influence infant feeding practices.

Finally, fathers classified as unresolved for loss or abuse were more likely than other fathers to exhibit controlling behavior during feeding, as hypothesized. The current results are also aligned with Messina’s et al.’s (2019) finding that higher scores for unresolved trauma on the AAI for mothers predicted higher levels of controlling behavior during feeding. Extending this connection between unresolved trauma and controlling feeding to fathers is important because multiple studies have found fathers to engage in more controlling feeding practices than mothers (Brann & Skinner, 2005; Powell et al., 2017; Pratt et al., 2017). Controlling parental feeding practices have the potential to interfere with children's developing ability to accurately perceive their own internal cues of hunger and satiety (Golan & Bachner-Melman, 2011; Patrick et al., 2013), which is especially important infancy when children are transitioning to solid foods, trying new foods, and beginning to experiment with self-feeding. Controlling feeding practices can take multiple forms (Vaughn, 2016) and different types of controlling feeding practices in fathers have been linked to different types of eating behavior difficulties in preschoolers, including food avoidance and food approach (Vollmer et al., 2015). Controlling feeding practices are important to understand because of the complex implications they have for child eating outcomes (e.g., Birch et al., 2003; Blissett et al., 2010; Golan & Bachner-Melman, 2011; Vollmer et al., 2015) as well as behavior problems as early as 2 years (Hemmi et al., 2011) and anxiety at age 7 (Messina, 2016). This study's findings indicate that unresolved attachment representations could be a key factor underlying this caregiver behavior. Future research should continue to explore factors that predict controlling caregiver feeding practices.

Fathers in this study were more controlling with their sons than their daughters, regardless of attachment representations. Prior research has found that fathers pressure their sons to eat more than their daughters (Orrell Valente, 2007), suggesting that fathers may relate to their sons and daughters differently in the context of feeding. Although the average size of infants does not actually differ by sex, fathers may be more likely to perceive boys as too small, based on gender stereotypes (Holm-Denoma et al., 2005). Such perceptions, combined with fathers’ strong focus on getting their child to eat (Khandpur et al., 2014), could drive fathers to want their sons to eat more to grow larger and stronger, potentially resulting in more controlling feeding behavior. Some data suggest fathers can be more involved with sons than with their daughters in infancy (NICHD Early Child Care Research Network, 2000), which could contribute to an increased desire to maintain control over their sons during feeding. The influence of both parent and child sex on feeding practices and behaviors should continue to be empirically explored.

### 4.1 Limitations and future directions

While this study had distinctive strengths, there were also notable limitations that future research should address. First, this study adapted Chattoor’s (1997) scale for use with our sample, which means that the results are not directly comparable to the results of other studies that used the original Chattoor scale. In addition, the variance accounted for by our models was significant but still relatively small, leaving questions open about other influential factors. For example, this study did not include assessments of fathers' own dietary practices or weight status. A recent study found that fathers who were overweight exhibited higher levels of controlling behavior with their young children than obese non-overweight fathers (Wendt et al., 2015). Additionally, paternal dietary intake has been found to predict child consumption (Harris & Ramsey, 2015). Future research should make a point of including measures of fathers' own weight status and eating behaviors to gain a more complete picture of factors influencing fathers' feeding practices.

The current sample was predominantly white, educated, and middle class. Only a handful of studies have examined paternal feeding practices in specifically non-white samples (e.g., Harris & Ramsey, 2015; Horodynsky & Arndt, 2005; Lora, Hubbs-Tait, Ferris, & Wakefield, 2016; Parada, Ayala, Horton, Ibarra, & Arredondo, 2016; Penilla et al., 2017), and these studies focused on current feeding practices and mealtime behavior, rather than understanding the roots of fathers feeding practices. Future studies should continue to explore feeding practices with fathers of diverse ethnic, socioeconomic, and educational backgrounds, ideally utilizing longitudinal designs to understand antecedents and predictors of fathers’ mealtime behavior with their children.

### 4.2 Implications for intervention

Numerous calls have been made to include fathers in parent feeding interventions to support the development of optimal feeding behaviors for all parents (Davison et al., 2016, 2018; Khandpur et al., 2014; Morgan et al., 2017; Watterworth et al., 2017). There has been limited father inclusion in obesity interventions, and an almost complete absence from prenatal interventions (Kotelchuck & Lu, 2017). This gap needs to be remedied, particularly in light of the present findings that fathers' feeding practices can be predicted prenatally, enabling interventions to take place even before infants' are born. Assessing first-time fathers’ attachment representations prenatally disentangles the effects of having a baby and past parenting experiences on their current feeding practices.

This study found that fathers' feeding practices may be influenced fathers' attachment representations, suggesting that interventions aimed at helping fathers develop healthy and attuned feeding practices could benefit from understanding that the feeding practices of fathers may be predisposed by their attachment representations. A key goal of such intervention should be to increase fathers' attuned and responsive feeding behaviors, particularly for dismissing fathers, who were found to be lower than other fathers on this dimension. In addition, fathers with histories of unresolved trauma may benefit particularly from interventions aimed at reducing controlling feeding patterns. Overall, study findings hold meaningful implications for developing interventions that account for fathers’ psychology and its link to behavior, and signal the potential value of preventative implementation of intervention as early as the prenatal period. Fathers are capable caregivers and their attachment histories influence their caregiving behaviors, including feeding, and this knowledge can strengthen both existing and future interventions, offering a new avenue of understanding and intervening to support optimal outcomes for child and family health.

## Funding

Funding acknowledgement: This research was made possible by a Medical Humanities New Investigator Award from the Wellcome Trust (Grant WT103343MA).
